# Intubation Versus Tracheotomy Outcomes in Critically Ill COVID-19 Patients in Low-Resource Settings: What Do We Know?

**DOI:** 10.3390/jcm14030978

**Published:** 2025-02-03

**Authors:** Pedja Kovacevic, Goran Baric, Sasa Dragic, Danica Momcicevic, Biljana Zlojutro, Milka Jandric, Tijana Kovacevic, Daniel Lovric, Ivan Palibrk, Jihad Mallat

**Affiliations:** 1 Medical Intensive Care Unit, University Clinical Centre of the Republic of Srpska, Dvanaest beba bb, 78000 Banja Luka, Bosnia and Herzegovina; goran.baric@kc-bl.com (G.B.); sasa.dragic@kc-bl.com (S.D.); danica.momcicevic@kc-bl.com (D.M.); milka.jandric@kc-bl.com (M.J.); 2 Faculty of Medicine, University of Banja Luka, Save Mrkalja 14, 78000 Banja Luka, Bosnia and Herzegovina; tijana.kovacevic@kc-bl.com; 3 Department of Pharmacy, University Clinical Centre Republic of Srpska, 78000 Banja Luka, Bosnia and Herzegovina; 4 Cardiology Clinic, University Hospital Center Zagreb, 10000 Zagreb, Croatia; daniel@dlovric.net; 5 Department of Anesthesiology, Reanimatology and Intensive Care, Clinic for Abdominal Surgery, University Clinical Centre of Serbia, 11000 Belgrade, Serbia; ivanpalibrk@yahoo.com; 6 Division of Critical Care Medicine, Critical Care Institute, Cleveland Clinic Abu Dhabi, Al Maryah Island, Abu Dhabi 112412, United Arab Emirates; 7 Cleveland Clinic Lerner College of Medicine, Case Western Reserve University, Cleveland, OH 44106, USA

**Keywords:** percutaneous tracheotomy, COVID-19, ARDS, mechanical ventilation, ventilator-free day

## Abstract

**Background**: Patients undergoing prolonged mechanical ventilation commonly require tracheotomy. The main aim of this study was to evaluate the outcomes of tracheotomy for patients with acute respiratory distress syndrome (ARDS) associated with COVID-19 in low-resource settings. **Methods**: A retrospective, single-center, observational cohort study was performed on patients with ARDS associated with COVID-19. Patients who underwent intubation alone were compared with those who received both intubation and subsequent tracheotomy. The analysis included patient demographics, comorbidities, and outcomes. **Results**: Patients undergoing tracheotomy (*n* = 89) were compared with intubated patients (*n* = 622). The median time from intubation to tracheotomy was 10 days (IQR: 6–15 days). Overall, 608 patients (85.5%) died in the hospital. Thirty-seven patients (35.9%) in the survival group had tracheostomy compared with fifty-two patients (8.5%) in the non-survival group (*p* < 0.001). The Kaplan–Meier curve shows a higher probability of survival in the tracheotomy group compared with the non-tracheotomy group (log-rank test: *p* < 0.001). Tracheotomy was found to be independently associated with lower in-hospital mortality (HR = 0.16 [95% CI: 0.11–0.23], *p* < 0.001) in the multivariable Cox proportional hazards regression analysis after adjusting for potential confounding factors. Furthermore, tracheotomy was associated with a higher cumulative incidence of being alive and off the ventilator at day 28 (SHR = 2.87 [95% CI: 1.88–4.38], *p* < 0.001). **Conclusions**: Tracheotomy was associated with reduced in-hospital mortality and longer ventilator-free days.

## 1. Introduction

Acute respiratory distress syndrome (ARDS) is a condition that develops rapidly (within seven days) and is characterized by inflammatory lung injury affecting the parenchyma of both lungs diffusely (bilateral lung infiltrates). The clinical hallmark of ARDS is hypoxia and non-cardiogenic pulmonary edema [1]. The latest (Berlin) definition categorizes ARDS into mild, moderate, and severe forms, with severe cases exhibiting a high mortality rate, reaching up to 45%. Mechanical ventilation is the mainstay of treatment for these patients, and the fact that 25% of all mechanically ventilated patients in the ICU suffer from ARDS underscores the significance of this issue [2,3]. The emergence of COVID-19 and its pandemic led to an exceptionally high number of patients experiencing ARDS associated with COVID-19 worldwide. Mortality from ARDS associated with COVID-19 ranged up to 80% in patients treated in low-resource settings [4,5]. On the other hand, compared with other viral infections such as influenza, increased rates of mechanical ventilation were reported in patients with COVID-19 [6]. The increasing severity of this type of ARDS often leads to prolonged weaning. Tracheotomy is recommended for all patients who require prolonged mechanical ventilation and do not meet the criteria for weaning. By undergoing a tracheotomy, the need for sedation and analgesia would likely be reduced, the weaning time would be shortened, and consequently, the ventilator-free days would increase [7]. The preferred method of tracheotomy in the critically ill is percutaneous dilatational tracheotomy compared with open surgical tracheotomy due to the lower percentage of complications, particularly infections of the operative site. During the COVID-19 pandemic, percutaneous dilatational tracheotomy was also the preferred technique because, in addition to the aforementioned advantages, it minimized hypoxia and aerosolization in patients with COVID-19 [8,9,10,11,12]. However, the clinical benefit of tracheotomy during the COVID-19 outbreak is not fully understood. Some earlier studies suggest that tracheotomy may be associated with reduced overall mortality rates [13,14,15]. However, tracheotomy indications and timing remain controversial [15,16,17,18]. When it comes to the treatment of critically ill individuals, both in general and those affected by COVID-19, reports from low-resource countries are scarce and inconsistent. The challenges faced by countries classified as low-resource settings in the field of intensive care medicine are multifaceted. These environments often suffer from a shortage of life-support equipment; even when such equipment is available, it is frequently non-functional. This paradox can be explained by the fact that, despite equipment procurement, the lack of officially trained medical personnel remains a critical issue. The COVID-19 pandemic clearly highlighted this, as mortality rates in these settings surged to as high as 80%. The noticeable shortage of this category of healthcare workers ultimately led to extremely challenging and sometimes nearly impossible care for intubated patients [4,19,20,21]. From all the above, it is evident why comparing intubation with tracheotomy in these patients within low-resource settings is significant. Given this lack of understanding, the benefits of tracheotomy in severe COVID-19 remain ambiguous, and more information is needed to best establish the clinical management guidelines. This study aimed to evaluate whether percutaneous dilatational tracheotomy was independently associated with hospital mortality and assess its impact on the number of ventilator-free days (VFDs) within the first 28 days of intensive care unit admission in low-resource settings.

## 2. Materials and Methods

### 2.1. Study Design, Setting, Population

This retrospective observational study, including all mechanically ventilated COVID-19 patients, was conducted at the largest medical intensive care unit in Bosnia and Herzegovina from 1 April 2020 to 1 January 2022. This study was approved by the Institutional Ethics Committee of the University Clinical Center of the Republic of Srpska (REC number: 01-19-191-2\22, on 19 April 2022) and the need for informed consent was waived due to the study’s retrospective nature. This medical intensive care unit is a reference center for treating all critically ill patients in the Republic of Srpska (an entity in Bosnia and Herzegovina), with a population of about 1,000,000 [19,20].

This study included all patients older than 18 who developed ARDS associated with COVID-19 and were admitted to the medical intensive care unit, meeting the inclusion criteria. Nasopharyngeal swabs and respiratory secretions were analyzed using reverse transcription polymerase chain reaction (RT-PCR). The inclusion criteria were as follows: age over 18 years, confirmed diagnosis of COVID-19 infection via a positive RT-PCR test, and intubated, mechanically ventilated patients with the presence of radiological signs of ARDS according to the Berlin criteria [1]. The exclusion criteria applied to all patients who underwent tracheotomy in an elective setting and those with an existing tracheostomy that required replacement. During the first year of the pandemic, only a very small number of patients underwent tracheotomy due to concerns about the potential spread of infection during the procedure. However, after that period, patients who underwent tracheotomy were selected based on the clinical judgment of the physicians.

### 2.2. Percutaneous Tracheotomy Procedure

Tracheotomy was performed according to the standard operating procedure of dilatative tracheotomy at the bedside using the Portex Percutaneous Tracheostomy Kit (ICU Medical), employing the Griggs technique. This technique, developed by Griggs et al. in 1990, is also known as the guidewire dilator forceps technique. The Portex Griggs percutaneous dilatational tracheotomy kit utilizes specially designed forceps (a modified Howard Kelly forceps) that slide over the guidewire to achieve a single-step dilation of the tissues in the paratracheal and tracheal spaces by spreading the forceps. After dilation, the tracheostomy tube is guided over the wire and inserted into the trachea. All procedures were always performed under bronchoscopy guidance, without exception, and they were carried out exclusively by experienced intensivist physicians [22,23]. Clinical data were obtained from the electronic patient records, and data collection ended with death or discharge from our ICU to either a rehabilitation facility, the referring hospital, the regular ward, or the home. The procedures followed the ethical standards of the responsible committee on human experimentation and the Helsinki Declaration of 1975.

### 2.3. Primary Outcome

The primary outcome was to investigate whether tracheostomy was independently associated with hospital mortality.

### 2.4. Secondary Outcome

The secondary outcome was the association between tracheostomy and the number of ventilator-free days (VFDs) during the first 28 days, defined as the number of days alive and free from mechanical ventilation for at least 48 consecutive hours [24]. Patients discharged from the hospital before 28 days were considered alive and free from mechanical ventilation at 28 days. Non-survivors at day 28 were considered to have no VFDs. For patients who died, the number of VFDs was 0. For patients who were alive, the VFDs were the days they did not require mechanical ventilation. For patients who required mechanical ventilation for more than 28 days, the number of VFDs was 0.

### 2.5. Statistical Analysis

The normality of data distribution was assessed using the Shapiro–Wilk test, and each variable’s distribution (histogram) was visually checked. Data are expressed as mean ± SD when normally distributed or as median [IQR] when non-normally distributed. Proportions were used as descriptive statistics for categorical variables. Comparisons of values between independent groups were performed with the two-tailed Student’s *t*-test or the Mann–Whitney U test, as appropriate. Analysis of the discrete data was performed by χ^2^ test or Fisher exact test when the numbers were small. There were missing data (missing at random) for malignancy (3.7%), time from symptoms to hospital admission (6.3%), lymphocyte (9.1%), neutrophile (9.1%), procalcitonin (3.1%), D-dimer (1.7%), LDH (2.5%), fibrinogen (2.4%), and lactate (2.0%) that were not imputed.

Time-to-event data were analyzed using the Kaplan–Meier method, and a log-rank test was used to compare hospital mortality of patients who had a tracheostomy and those who did not. Adjusted Cox proportional hazards regression analysis was used to investigate the association between tracheostomy and time to in-hospital death using clinically likely confounders including age, gender, SOFA/SAPS II scores, use of corticosteroids, and tocilizumab. In addition, variables associated with time to clinical improvement (*p* < 0.1) in univariate analysis were also included in the adjusted Cox model. The potential problem of co-linearity was evaluated using the Spearman or Pearson correlation coefficient before running the analysis. The proportionality hazard assumption was assessed using the Schoenfeld residuals. Hazard ratios (HRs) and 95% confidence intervals were summarized.

The secondary outcome (VFDs) was evaluated with competing-risks regression based on Fine and Gray’s proportional sub-hazards model. Death before day 28 was considered to be the competing event, and time-to-event analysis was right-censored at 28 days. Sub-hazard ratios (SHRs) and 95% confidence intervals were summarized. Also, to estimate the effects of tracheostomy on ventilator-free days, we used zero-inflated negative binomial regression analysis. The effect size was calculated as the mean difference and its respective 95% confidence interval.

A value of *p* < 0.05 was considered statistically significant, and all reported *p*-values are two-sided. Statistical analyses were performed using Stata 17.0 software for Windows (Stata Corp LLC, Collage Station, TX 77845, USA).

## 3. Results

### 3.1. Study Population

From 1 April 2020 to 1 January 2022, 711 adult patients with ARDS caused by COVID-19 infection requiring invasive mechanical ventilation were admitted to the ICU. Since 51.8% of the patients (*n* = 368) died within 10 days of ICU admission, with only 8 patients (1.1%) having tracheotomy by day 10, only patients intubated longer than 10 days (*n* = 343) were analyzed in this study. Table 1 summarizes the main characteristics of the cohort. The median age among all patients was 64 years (IQR: 56–71 years), and 183 patients (69.5%) were men. The median time from symptoms onset to hospital admission was 6 days (IQR: 4–8 days). The median time from hospital admission to ICU admission was 4 days (IQR: 1–7 days).

Eighty-one patients (23.6%) had tracheotomy during their ICU stay. The median time from intubation to tracheotomy was 11 days (IQR: 6–15 days). Patients’ age was significantly higher in the non-tracheotomy group than in the tracheotomy group (Table 1). However, comorbidities were not significantly different between the two groups. The SAPS II score was significantly higher in the non-tracheotomy group compared with the tracheotomy group. Also, the SOFA and SAPS II scores were not significantly different between the tracheotomy and non-tracheotomy groups (Table 1). Regarding laboratory data on ICU admission, lactate dehydrogenase, fibrinogen, and lactate levels were significantly higher in the non-tracheotomy group than in the tracheotomy group (Table 1). The incidence of AKI was not significantly different between the two groups. Tocilizumab, steroids, and renal replacement therapy treatments did not differ between the two groups. Cytosorb was used more in the tracheotomy group (Table 1). The incidence of pneumothorax was significantly higher in the tracheotomy group than in the non-tracheotomy group (Table 1). The median hospital length of stay was longer in the tracheotomy group compared with the non-tracheotomy group (31 days (IQR: 23–46 days) vs. 19 days (IQR: 15–24 days), *p* < 0.001). However, 29 patients (35.8%) in the tracheotomy group were discharged home compared with 25 patients (9.6%) in the non-tracheotomy group (*p* < 0.001).

### 3.2. Primary Outcome

Overall, 268 patients (78.1%) died in the hospital after 10 days of ICU admission. Table 2 presents the characteristics of patients between survival and non-survival groups. CKD, corticosteroid use, lactate dehydrogenase levels, lactate levels, AKI rate, and tracheotomy significantly differed between the survival and non-survival groups. Thirty-seven patients (49.3%) in the survival group had a tracheotomy compared with forty-four patients (16.4%) in the non-survival group (*p* < 0.001).

**Figure 1:**
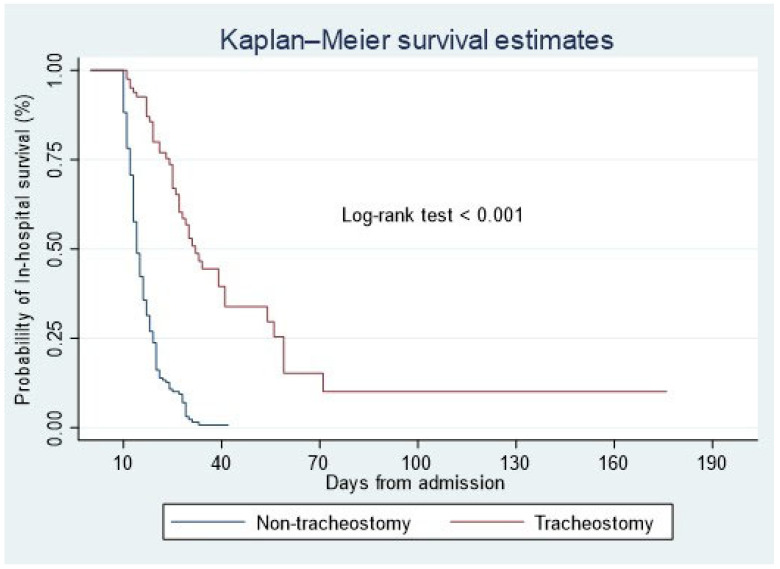
Kaplan–Meier survival curve shows the tracheotomy group had a significantly better probability of survival compared with the non-tracheotomy group.

The Kaplan–Meier curve shows significantly higher survival in the tracheostomy group compared with the non-tracheostomy group (log-rank test: *p* < 0.001) (Figure 1). The median survival time in the non-tracheostomy group was 14 days (95% CI: 14–15) compared with 32 days (95% CI: 27–41) (*p* < 0.001). The restricted mean survival time was 16.2 days (95% CI: 15.4–17.0) in the non-tracheostomy group compared with 49.3 days (95% CI: 33.1–65.6) in the tracheostomy group (*p* < 0.001).

**Figure 2:**
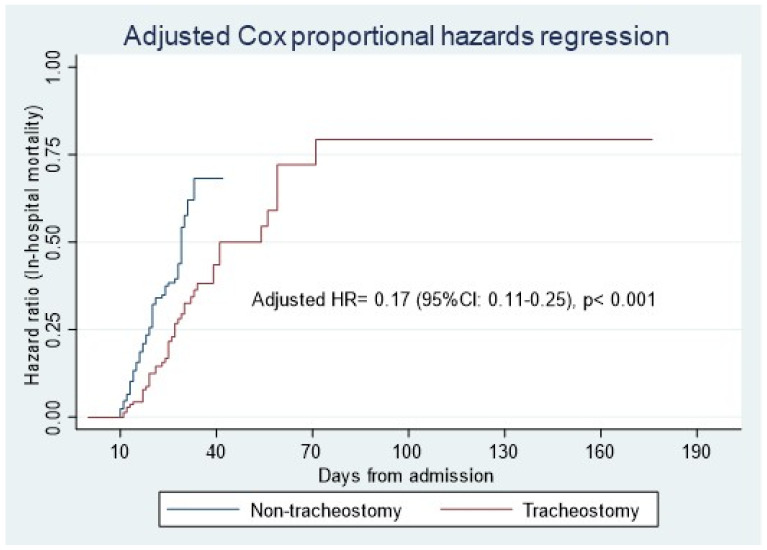
The adjusted Cox proportional hazards model shows lower in-hospital mortality hazards for the tracheostomy group. Adjusted for age, gender, SAPS II score, SOFA score, lactate level, lactic dehydrogenase level, acute kidney injury, tocilizumab, and corticosteroids. HR: hazards ratio.

In the multivariable Cox proportional hazards regression analysis, after adjusting for the above confounder variables and age, gender, SAPS II score, SOFA score, and tocilizumab, tracheostomy was found to be independently associated with lower in-hospital mortality (HR = 0.16 [95% CI: 0.11–0.23], *p* < 0.001) (Table 3) (Figure 2).

### 3.3. Secondary Outcome

**Figure 3:**
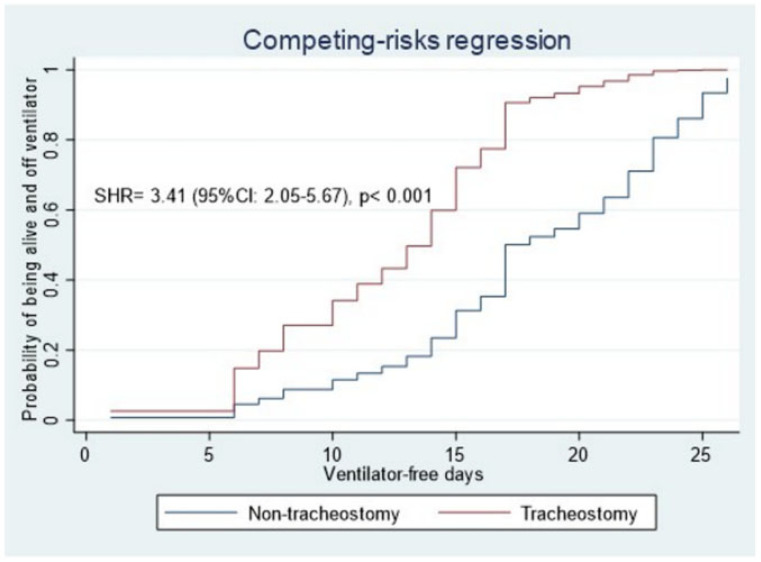
Cumulative incidence functions for ventilator-free days in patients with tracheostomy and those without. The probability of being alive and off the mechanical ventilator was significantly higher in the tracheostomy group than in the non-tracheostomy group. SHR: sub-hazards ratio.

In the competing-risks regression analysis, tracheostomy was associated with a higher cumulative incidence of being alive and off the ventilator at day 28 (SHR = 3.41 [95% CI: 2.05–5.67], *p* < 0.001) (Figure 3).

**Figure 4:**
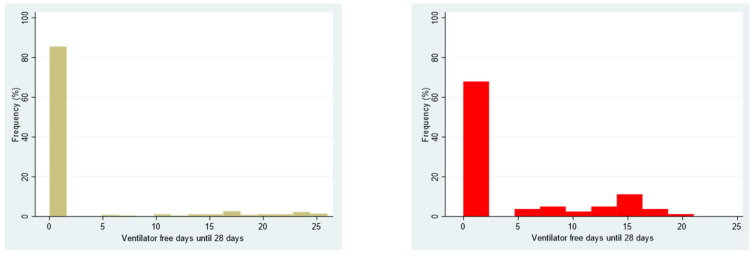
Distribution of ventilator-free days in the non-tracheostomy group (**A**) and tracheostomy group (**B**).

The mean number of days alive and free from mechanical ventilation during the first 28 days was significantly higher in the tracheostomy group than in the intubated group (4.0; 95% CI, 2.6–5.4 days vs. 2.6; 95% CI, 1.8–3.4 days; difference, 1.50; 95% CI, −0.11–3.06; *p* = 0.07) (Figure 4).

## 4. Discussion

The main findings of this study were that PDT was significantly and independently associated with lower in-hospital mortality after adjusting for potential confounders, and percutaneous dilatational tracheotomy was associated with longer ventilator-free days.

Data sources in the literature for the population with ARDS associated with COVID-19 who underwent tracheotomy are available, but knowledge is limited, especially in low-resource settings. Given the significant differences in healthcare systems, a regional analysis may prove beneficial, particularly in the Western Balkans area of Southeast Europe. To our knowledge, this is the only study conducted in this area that examines the characteristics and outcomes of critically ill COVID-19 patients who underwent percutaneous dilatational tracheotomy in low-resource settings. Tracheotomy is a procedure recommended for critically ill patients requiring long-term mechanical ventilation.

The results of this study suggest that percutaneous dilatational tracheotomy was associated with reduced in-hospital mortality among critically ill COVID-19 patients, which aligns with findings from other authors. Reduced in-hospital mortality among critically ill COVID-19 tracheotomized patients was demonstrated in previous studies [15,25,26,27,28]. Indeed, in a retrospective multicenter study performed in Israel that included 157 critically ill COVID-19 patients, among whom 30 patients (19.1%) underwent tracheotomy, Rozenblat et al. found that tracheotomy was significantly associated with longer survival in multivariable analysis after adjusting for potential confounders (odds ratio: 0.37, *p* = 0.004) [15]. In that study, the median time from ICU admission to performing tracheotomy was 13 days [15]. In another retrospective study that included 258 critically ill, mechanically ventilated COVID-19 patients, among whom 46 patients (18%) had a tracheostomy, Molin et al. reported a higher mortality rate in the non-tracheostomy group compared with the tracheostomy group (54% vs. 29%, respectively, *p* < 0.01) [25]. However, no multivariable analysis was performed in that study. The median time on mechanical ventilation before tracheotomy was 14 days [25]. Furthermore, in a retrospective cohort multicenter study from the US, Alnemri et al. included 777 mechanically ventilated COVID-19 patients, with 185 patients (23.8%) having tracheostomy [26]. The authors found that tracheostomy was significantly associated with lower mortality using multivariable analysis (OR: 0.31, *p* < 0.001). Moreover, in a prospective multicenter cohort study performed in Italy, Corona et al. included 248 critically ill COVID-19 patients, with 128 patients (51.6%) undergoing tracheotomy [27]. Tracheotomy was performed after a median of 11 days. The authors observed that the cumulative survival was higher in patients who had tracheotomy than those who did not (log-rank test = 4.8, *p* = 0.028) [27]. Also, in a retrospective study from Chile that included 92 mechanically ventilated COVID-19 patients, among whom 46 patients (50%) underwent tracheotomy, the authors found a lower mortality rate in the patients who were tracheotomized than in the non-tracheotomy group (6.5% vs. 32.6%, *p*-value < 0.01) [28]. However, when adjusting for confounding variables in a multivariable analysis, no significant differences were observed in mortality between the two groups [28]. Nevertheless, the longer time to perform tracheotomy in that study (median of 20.5 days) [28] could explain the discrepancy between these findings and our results, since the median time to tracheotomy was 10 days in our study. On the other hand, findings are contradictory regarding the optimal timing for tracheotomy (early versus late tracheotomy) [15,25,26,27,28]. A recent meta-analysis of 47 studies investigating the impact of tracheotomy timing and technique on COVID-19 mortality found that while tracheotomy significantly reduced mortality, its timing did not influence mortality rates, the duration of mechanical ventilation, or the time to decannulation [29]. These findings support our study’s results, which identified an inverse relationship between tracheotomy and in-hospital mortality.

The findings of this study indicate that tracheotomized patients had longer ventilator-free days, at 28 days. Similar results were observed by other authors. In the study by Hernandez et al., early tracheotomy was significantly associated with more ventilator-free days within the first 28 and 60 days post-intubation as well as a higher number of ICU- and hospital-free days in the same timeframes following admission. Furthermore, these findings suggest that earlier tracheotomy improves patient prognosis and optimizes ICU resource availability [30]. In the group of tracheotomized patients, there was a statistically significant increase in cases of pneumothorax. This result may be attributed to the statistically longer hospital stays observed in this group, as prolonged hospitalization is associated with a higher incidence of pneumothorax and pneumomediastinum [31]. The significant number of pneumothoraxes in patients who underwent tracheotomy did not result from periprocedural causes, as no complications during the procedure were recorded in any of the observed patients. On the other hand, numerous studies demonstrate that pneumothorax can occur spontaneously in COVID-19 patients, even in non-mechanically ventilated and previously completely healthy patients. In addition, the implementation of up-to-date therapeutic and diagnostic procedures, including training in percutaneous dilatational tracheotomy, is feasible in low-resource settings. This feasibility is demonstrated by experiences from Bosnia and Herzegovina [19,20,21].

The mean age of patients in this study was 65 years, with the majority being male, who also can experience poorer treatment outcomes. These findings align with results from other studies [32,33,34]. A high mortality rate among invasively mechanically ventilated COVID-19 patients is also characteristic of countries classified as low-resource settings in terms of intensive care medicine, which includes the countries of the Western Balkans [35]. The term “low-resource settings” refers to countries or environments that lack the necessary resources to provide adequate treatment for those who are critically ill [5]. The definition of low-resource settings is used to describe healthcare systems in low-income countries, low- to middle-income countries, and upper-middle-income countries, recognizing that low-resource settings can also be present in high-income countries [4,35]. The high mortality rate among invasively mechanically ventilated patients in this study is consistent with findings from other authors in low-resource settings [4,35]. The COVID-19 pandemic and the many critically ill patients underscored the importance of developing modern intensive care medicine in low-resource settings. The fact that more than two-thirds of the world’s population lives in low-resource-settings countries makes this issue even more significant [36].

Finally, it is necessary to summarize all the positive effects of tracheotomy that might have contributed to a higher survival rate in this group of patients. The cumulative sedation dose will certainly decrease after the tracheotomy is performed, and these patients begin physical therapy and rehabilitation significantly earlier. Early mobilization and physical therapy reduce the likelihood of venous thromboembolic complications in these patients. Tracheotomy in patients requiring prolonged ventilation leads to earlier weaning from the ventilator as well as earlier walking, eating, and speaking. Finally, tracheotomy reduces the risk of complications that are seen after trans-laryngeal intubation, such as tracheomalacia and tracheal stenosis [37,38].

In light of its limitations, it is prudent to approach the outcomes of this study with care. The most important among these limitations is the small number of patients who received tracheotomy, a factor known to diminish study robustness. One of the limitations that should be noted is that during periods of ICU overcrowding, when occupancy exceeded 80%, the number of tracheotomies performed was significantly lower. On the other hand, under these conditions and among these patients, mortality was significantly higher. Additionally, the study’s confinement to a single center poses another significant limitation, potentially curtailing its broader generalizability to other centers. In addition to the above, the retrospective, non-randomized cohort design introduces potential biases. However, we performed a multivariable analysis and adjusted for several potential confounders. Also, the absence of long-term follow-up for the study population should be considered. However, as can be seen from Figure 1 and Figure 2, the maximum follow-up was 175 days. Future research directions can also be highlighted.

## 5. Conclusions

In conclusion, it is important to highlight several significant findings from this single-center study. Firstly, it was observed that COVID-19 patients with ARDS require prolonged mechanical ventilation and show significantly lower in-hospital mortality rates when undergoing percutaneous dilatational tracheotomy. In addition to lowering hospital mortality, percutaneous dilatational tracheotomy was associated with an increased number of ventilator-free days in the first 28 days. However, to our knowledge, this is the first study conducted in the region of the Balkan countries. Consequently, further research is necessary to validate these findings.

## Figures and Tables

**Table 1:** Comparisons of baseline characteristics, laboratory data, and treatments between tracheotomy and non-tracheotomy groups.

	All Patients (*n* = 343)	Non-Tracheotomy (*n* = 262)	Tracheotomy (*n* = 81)	*p* -Value
**Age, y**	64 [56–71]	64 [57–72]	64 [53–69]	0.160
**Male, *n* (%)**	230 (67.1)	183 (69.5)	47 (58.0)	**0.048**
**Diabetes, *n* (%)**	103 (30.0)	81 (30.9)	22 (27.2)	0.52
**Chronic heart failure, *n* (%)**	154 (44.9)	122 (46.6)	32 (39.5)	0.26
**Chronic lung disease, *n* (%)**	18 (5.2)	9 (3.4)	9 (11.1)	**0.02**
**Chronic liver disease, *n* (%)**	8 (2.3)	7 (2.7)	1 (1.2)	0.69
**Chronic kidney disease, *n* (%)**	22 (6.4)	13 (5.0)	9 (11.1)	0.07
**Obesity, *n* (%)**	42 (12.2)	33 (12.6)	9 (11.1)	0.85
**Rheumatologic disease, *n* (%)**	16 (4.7)	13 (5.0)	3 (3.7)	0.77
**Hypothyroid, *n* (%)**	19 (5.5)	16 (6.1)	3 (3.7)	0.58
**Malignancy, *n* (%)**	15 (4.6)	12 (4.8)	3 (3.7)	1.00
**Time from symptoms to hospital admission, days**	6 [4–8]	6 [4–8]	6 [3–7]	0.21
**Time from hospital to ICU admission, days**	4 [1–7]	4 [1–7]	4 [1–6.5]	0.57
**SOFA score**	4 [2–7]	4 [2–7]	4 [2–7]	0.84
**SAPS II score**	27 [18–39]	28 [19–39]	24 [18–37]	0.11
**Tocilizumab, *n* (%)**	14 (4.1)	11 (4.2)	3 (3.7)	1.00
**Corticosteroids, *n* (%)**				0.39
**Methylprednisolone**	283 (82.5)	220 (84.0)	63 (77.8)	
**Dexamethasone**	44 (12.8)	31 (11.8)	13 (16.0)	
**None**	16 (4.7)	11 (4.2)	5 (6.2)	
**C-reactive protein, mg/L**	96.1 [43.8–169.0]	100.0 [43.8–171.0]	87.0 [45.0–147.0]	0.44
**Procalcitonin, ng/mL**	0.2 [0.1–0.6]	0.2 [0.1–0.6]	0.2 [0.1–0.7]	0.83
**Leucocyte count, ×10^9^/L**	11.6 [8.4–16.3]	11.7 [8.4–16.7]	11.5 [8.4–15.1]	0.54
**Lymphocyte count, ×10^9^/L**	0.65 [0.42–0.98]	0.63 [0.41–0.98]	0.71 [0.50–0.90]	0.26
**Neutrophil count, ×10^9^/L**	9.8 [6.6–13.7]	9.8 [6.6–13.7]	9.7 [6.5–13.9]	0.91
**D-dimer, µg/mL**	2.5 [1.2–10.6]	2.5 [1.1–10.0]	2.0 [1.2–11.7]	0.92
**Lactate dehydrogenase, U/L**	540 [413–695]	552 [441–701]	487 [324–674]	**0.04**
**Fibrinogen, g/L**	5.2 [3.9–6.1]	5.3 [3.8–6.2]	4.8 [3.9–5.5]	**0.04**
**Lactate, mmol/L**	2.0 [1.5–2.6]	2.0 [1.5–2.6]	1.8 [1.3–2.5]	**0.03**
**Acute kidney injury, *n* (%)**	110 (32.1)	86 (32.8)	24 (29.6)	0.59
**Continuous renal replacement therapy, *n* (%)**	62 (18.1)	44 (16.8)	18 (22.2)	0.27
**Cytosorb filter, *n* (%)**	15 (4.4)	5 (1.9)	10 (12.3)	**<0.001**
**Deep venous thromboembolism, *n* (%)**	7 (2.0)	7 (2.7)	0 (0.0)	0.20
**Pulmonary embolism, *n* (%)**	15 (4.4)	11 (4.2)	4 (4.9)	0.76
**Pneumothorax, *n* (%)**	22 (6.4)	11 (4.2)	11 (13.6)	**0.007**

SOFA, Sequential Organ Failure Assessment; SAPS, Simplified Acute Physiology Score; ICU, intensive care unit. Data are reported as median (IQR), mean ± SD, or number (proportion).

**Table 2:** Comparisons of baseline characteristics, laboratory data, and treatments between survival and non-survival groups.

	Non-Survival (*n* = 268)	Survival (*n* = 75)	*p* -Value
**Age, y**	64 [57–72]	64 [54–70]	0.37
**Male, *n* (%)**	178 (66.4)	52 (69.3)	0.63
**Diabetes, *n* (%)**	75 (28.0)	28 (37.3)	0.12
**Chronic heart failure, *n* (%)**	119 (44.4)	35 (46.7)	0.73
**Chronic lung disease, *n* (%)**	12 (4.5)	6 (8.0)	0.24
**Chronic liver disease, *n* (%)**	6 (2.2)	2 (2.7)	0.69
**Chronic kidney disease, *n* (%)**	11 (4.1)	11 (14.7)	**0.002**
**Obesity, *n* (%)**	35 (13.1)	7 (9.3)	0.43
**Rheumatologic disease, *n* (%)**	13 (4.8)	3 (4.0)	1.00
**Hypothyroid, *n* (%)**	14 (5.2)	5 (6.7)	0.58
**Malignancy, *n* (%)**	13 (5.1)	2 (2.7)	0.53
**Time from symptoms to hospital admission, days**	6 [4–8]	6 [4–10]	0.55
**Time from hospital to ICU admission, days**	4 [1–7]	3 [1–7]	0.58
**SOFA score**	4 [2–7]	4 [3–8]	0.18
**SAPS II score**	27 [18–38]	28 [18–39]	0.97
**Tocilizumab, *n* (%)**	11 (4.1)	3 (4.0)	1.00
**Corticosteroids, *n* (%)**			**0.02**
Methylprednisolone	227 (84.7)	56 (74.7)	
Dexamethasone	33 (12.3)	11 (14.7)	
None	8 (3.0)	8 (10.7)	
**C-reactive protein, mg/L**	97 [43–169]	85 [45–173]	0.71
**Procalcitonin, ng/mL**	0.2 [0.1–0.6]	0.2 [0.1–0.7]	0.34
**Leucocyte count, ×10^9^/L**	11.5 [8.3–16.2]	11.9 [8.5–16.7]	0.32
**Lymphocyte count, ×10^9^/L**	0.64 [0.42–0.94]	0.67 [0.41–1.08]	0.58
**Neutrophile count, ×10^9^/L**	9.7 [6.5–13.7]	10.2 [7.4–13.6]	0.32
**D-dimer, µg/mL**	2.5 [1.1–10.6]	2.5 [1.2–10.6]	0.90
**Lactate dehydrogenase, U/L**	554 [441–701]	479 [309–680]	**0.006**
**Fibrinogen, g/L**	5.3 [3.9–6.2]	5.0 [3.9–5.7]	0.25
**Lactate, mmol/L**	2.0 [1.5–2.7]	1.8 [1.3–2.2]	**0.003**
**Acute kidney injury, *n* (%)**	101 (37.7)	9 (12.0)	**<0.001**
**Continuous renal replacement therapy, *n* (%)**	49 (18.3)	13 (17.3)	0.85
**Cytosorb filter, *n* (%)**	14 (5.3)	1 (1.3)	0.21
**Deep venous thromboembolism, *n* (%)**	5 (1.9)	2 (2.7)	0.65
**Pulmonary embolism, *n* (%)**	13 (4.8)	2 (2.7)	0.54
**Pneumothorax, *n* (%)**	19 (7.1)	3 (4.0)	0.43
**Tracheotomy, *n* (%)**	44 (16.4)	37 (49.3)	**<0.001**

SOFA, Sequential Organ Failure Assessment; SAPS, Simplified Acute Physiology Score; ICU, intensive care unit. Data are reported as median (IQR), mean ± SD, or number (proportion).

**Table 3:** Factors associated with in-hospital mortality (multivariable Cox regression analysis).

Variable	Hazards Ratio (95% Confidence Interval)	*p* -Value
**Tracheostomy (reference: non-tracheostomy)**	0.17 (0.11–0.25)	**<0.001**
**Age, years**	1.02 (1.00–1.03)	**0.006**
**SAPS II score**	0.99 (0.98–1.00)	0.19
**SOFA score**	1.02 (0.98–1.07)	0.25
**Lactate**	1.01 (1.00–1.02)	**0.03**
**Acute kidney injury**	0.91 (0.78–1.14)	0.23
**Lactate dehydrogenase**	1.00 (0.99–1.00)	0.06
**Corticosteroids**	1.01 (0.77–1.34)	0.92
**Tocilizumab**	1.00 (0.53–1.90)	0.99
**Gender**	1.11 (0.85–1.46)	0.43

SOFA, Sequential Organ Failure Assessment; SAPS, Simplified Acute Physiology Score. Goodness-of-fit test (Schoenfeld): *p* = 0.6117.

## Data Availability

The data presented in this study are available on request from the corresponding author. The data are not publicly available due to the Ethics Committee restrictions.

## Abbreviations

ICU: intensive care unit; ARDS, acute respiratory distress syndrome; VFD, ventilator-free day; COVID-19, corona virus infectious disease 2019; SOFA, Sequential Organ Failure assessment; SAPS, Simplified Acute Physiology Score.
